# Problematic Internet Use and Its Relationship with Cyberbullying, Anxiety, and Executive Functions in Adolescence

**DOI:** 10.3390/children12040503

**Published:** 2025-04-14

**Authors:** Marta Real-Fernández, Ignasi Navarro-Soria, Megan Rosales-Gómez, Beatriz Delgado

**Affiliations:** Department of Developmental Psychology and Didactics, Faculty of Education, University of Alicante, 03690 Alicante, Spain; marta.real.fernandez@gmail.com (M.R.-F.); meganrosales37@gmail.com (M.R.-G.); beatriz.delgado@ua.es (B.D.)

**Keywords:** Executive Functions, anxiety, cyberbullying, cybervictimization, Problematic Internet Use, adolescence

## Abstract

**Introduction/objectives:** Brain development changes during adolescence are directly linked to various cognitive and behavioral challenges characteristic of this stage. The main objective of this study is to investigate the risks associated with Internet use and its relationship with Executive Functions (EFs) and anxiety in a representative sample of Spanish secondary school students. **Methods:** The sample consisted of 1164 participants (48% males) aged 12 to 17 years (M age = 14.86; SD = 1.41) from five selected academic centers. Executive Functions were assessed using the Adolescent and Adult Executive Functioning Questionnaire (ADEXI), anxiety was measured with the Depression, Anxiety and Stress Scale (DASS-21), and Problematic Internet Use (PIU) was evaluated with the Internet Addiction Test (IAT). **Results:** Significant positive correlations were found between the PIU, EF, anxiety, and cyberbullying variables. Predictive models were developed to explain the different variables. **Conclusions:** The results emphasize the need to increase awareness of these issues and to develop effective intervention strategies. Programs that promote responsible Internet use, along with classroom activities addressing anxiety and Executive Functions, could provide clear benefits.

## 1. Introduction

Adolescence is a stage characterized by numerous changes, including psychological and emotional changes [1]. During this period, a social shift occurs in which peer relationships gain greater significance [2]. Although this phenomenon has always been present, its manifestation has varied across generations.

Currently, adolescence is accompanied by increasing use of Information and Communication Technologies (ICTs). About 92% of young people aged 10 to 15 are frequent Internet users [3]. While its use is widespread and brings numerous benefits, the literature indicates that inappropriate or excessive use may have significant psychological and behavioral consequences [4]. Although it occurs across all groups and sectors, its greater prevalence among adolescents raises more concerns about the potential negative consequences for this population. Problematic Internet Use (PIU) is at the core of these concerns, and it refers to a behavioral pattern characterized by a loss of control over Internet use, which leads to negative consequences in daily life, such as academic difficulties, physical and mental health problems, and conflicts in social and family relationships. This problematic use differs from normal use of social media or the Internet in that the former significantly interferes with an individual’s daily functioning, whereas the latter comprises use through balanced integration in adolescents’ lives without causing adverse effects [5]. As previously mentioned, adolescence is one of the most sensitive developmental periods [6]. With age, Internet use tends to normalize, becoming a tool used more frequently in professional settings and less for recreational purposes; adults are generally more aware of the problem and the negative effects of excessive use, and they tend to show greater self-regulation [7,8].

Another risk factor in PIU, in addition to age, is gender. Various authors have pointed out that although boys tend to spend more time on the Internet [9], it is girls who show more problematic patterns of use [4,10]. Although these findings remain controversial, given the many variables that must be considered, they are nonetheless consistent with the fact that girls are more prone to seeking approval and engaging in social comparison [11]. As a result, they experience a stronger emotional impact (such as anxiety and/or depression and damage to self-esteem and self-image) when using social media and messaging platforms. Boys, in contrast, tend to seek out challenge and competition, often through video games and using them as a means of social connection [11]. In this way, we observe that this phenomenon has led to a range of issues, among which cyberbullying can be found. In Spain, cyberbullying has been identified as one of the most prevalent forms of bullying, particularly among youth [12], and, in recent years, reports of cyber-related offences against minors, including cyberbullying, have shown a steady increase. This phenomenon has reached up to a 72% rate of prevalence [13]. Moreover, this growing concern is supported by recent evidence showing that Problematic Internet Use (PIU) is a significant predictor of changes in emotional well-being, externalizing behaviors, and Internet-related risky behaviors, including cyberbullying. One study [14] found that high levels of PIU predicted increases in both proactive and reactive aggressive behaviors, a decrease in positive emotions, and, consequently, a rise in cyberbullying, both in terms of cybervictimization and cyberaggression. These findings are consistent with previous research [15,16], suggesting that adolescents who spend more time online and use the Internet in maladaptive ways are more exposed to its risks, which can increase their involvement in cyberbullying dynamics.

This inappropriate use of ICT and its associated consequences are closely linked to brain development changes characteristic of this stage. One of the most affected elements is Executive Functions (EFs) [17], which enable intentional and deliberate interactions with the world. They include, but are not limited to, attention and concentration, impulse control, decision making, task monitoring, and working memory [18]. These functions serve as essential tools in daily activities that form the foundation of cognitive, psychological, and social development. They emerge from the development of the prefrontal cortex and its connections with other brain regions, beginning in infancy and continuing through adolescence and early adulthood. They are shaped by environmental factors, such as stress, sleep, physical health, and brain plasticity [19]. A study [20] assessed whether there are differences between inhibitory control and cognitive flexibility among victims, perpetrators, bully victims, and witnesses of bullying, and their findings revealed differences in cognitive flexibility, which was lower in victims, but no significant differences in inhibitory control.

Similarly, several studies have investigated the relationship between EFs and psychological symptoms in adolescence, given that, as mentioned earlier, this stage is typically marked by an increased prevalence of emotional difficulties [21,22] It has been found that good use of EFs is associated with a better ability to process emotional information, thereby enabling the capacity to confront personal and social situations with better resilience and coping skills [23]. Consequently, these cognitive abilities also help reduce anxious and depressive symptoms, as they allow individuals to identify and regulate self-destructive thoughts on an emotional level [24]. Regarding externalizing behaviors, research has shown [25] that aggression in children is negatively associated with executive functioning; thus, young people with a prosocial attitude are better able to recall and apply assertive strategies in situations involving anger. These individuals, possessing stronger EFs, may be more capable of inhibiting aggressive responses [26]. The authors also found a bidirectional relationship between peer conflict and EFs in childhood and adolescence, as children and adolescents involved in more interpersonal conflicts tended to exhibit poor functioning.

Considering these findings, deficits in EFs, particularly poor working memory, planning, and inhibitory control, can be understood as potential individual risk factors that could predict and explain the mechanisms underlying aggressive behaviors during this developmental stage [27,28]. The literature extensively reports an inverse relationship between EFs and aggressive behavior [29,30,31,32]. For instance, a study conducted with preschool children demonstrated that higher scores related to behavioral problems coincided with lower performance on EF assessments [33]. Furthermore, a systematic review [34] analyzed multiple studies proposing that Executive Functions should be considered key factors for understanding the individual characteristics underlying aggressive behavior, which consequently sustain bullying dynamics. Among the reviewed studies, those of [25,34,35,36,37,38,39,40] stood out, determining that EFs (working memory, shifting, and inhibition) correlated significantly with various forms of aggression and played a crucial role in regulating this type of behavior. Inhibitory control is fundamental because it allows individuals to regulate inappropriate behaviors in specific contexts and respond appropriately to complex situations, thereby facilitating effective adaptation to constantly changing environments and contributing to the prevention of behavioral problems [41]. Likewise, working memory, along with cognitive flexibility, also represents one of the core processes and plays a critical role in resolving social conflicts [42].

Furthermore, using the brief EF questionnaire Webexec with adolescents from Spain, one study [43] aimed to analyze whether Webexec correlated with internalizing symptoms, such as anxiety and aggressive behavior. Their findings indicated a positive correlation between EFs and these symptoms. Another study obtained similar results, particularly highlighting a greater difference in the state of anxiety [44]. Furthermore, in another study, the findings not only reinforced the relationship between EFs and manifestations of anxiety and/or depression but also emphasized its importance in the educational context [45]. Consistent with this evidence, other studies have linked anxiety to different EF components, such as inhibition, cognitive flexibility, working memory, organization, categorizing, planning, attention, verbal fluency, decision making, initiative, and emotional regulation, among others. According to [46], there is an inverse correlation between anxiety and inhibitory capacity. However, they found no significant relationship between anxiety and other EF components. In contrast, another study [47] aimed to determine whether anxiety symptoms were associated with dysexecutive symptomatology and found a significant positive correlation between the two. A different study analyzed the relationship between levels of anxiety in adults and their capacity for problem resolution, concluding that the subjects with higher levels of anxiety exhibited more difficulties in problem resolution tasks compared to those with lower anxiety levels [48].

On the other hand, given the strong relationship between these psychological processes, their impact on social media and Internet use can be easily understood. A conducted study [49] analyzed the relationship between PIU and Executive Functions, specifically inhibitory control, planning, and goal achievement. The results showed that lower inhibitory control was associated with PIU and was more frequent in the older age group. Likewise, its relationship to psychopathological mechanisms has been described [50]. A study reviewed the psychological and physical variables that could be associated with PIU and found that anxiety was among them [51]. Similarly, [52] confirmed a significant and direct correlation between PIU and social anxiety, as well as obsessive–compulsive responses. As previously mentioned, one of the consequences of PIU is the emergence of issues like cyberbullying. Some authors, such as Yudes-Gómez et al. [53], have identified PIU as a strong predictor of both cyberbullying and cybervictimization. Through a study of a large sample of primary and secondary school students, an 8% prevalence of cyberbullying was found, along with high levels of anxiety, aggressiveness, and shifts in interests related to problematic ICT use among both perpetrators and victims compared to non-involved individuals [54].

Based on the review of the current literature, the objectives of this study are defined as focusing on exploring the relationships between various psychological and behavioral factors relevant to adolescence. The main objective is to investigate the risks associated with Internet use and its relationship with Executive Functions and anxiety in a representative sample of secondary school students (aged 12 to 17). The specific objectives include examining the relationship between three of these phenomena, PIU, cybervictimization, and cyberbullying, and the previously mentioned variables. It is expected that greater difficulties in EFs and higher levels of anxiety may be linked to increased vulnerability to these factors. Accordingly, the hypotheses proposed are as follows. (H1) There will be a higher likelihood of PIU among students with EF difficulties, high anxiety levels, and a history of experiencing or perpetrating cyberbullying. (H2) Students will be more likely to suffer cybervictimization when they present EF deficits and high anxiety levels and engage in PIU. (H3) A higher likelihood of committing cyberbullying will be associated with EF deficits, high anxiety levels, and PIU.

## 2. Materials and Methods

### 2.1. Participants

The sample consisted of 1164 participants (560 males and 604 females). A convenience sampling method was used, as access was readily available to students in the 7th to 11th grades (*Educación Secundaria Obligatoria* and *1° de bachillerato* in Spain) in the province of Alicante (Spain). According to official data from the *Instituto Nacional de Estadística* (INE), the total population of adolescents aged 12 to 17 in the province of Alicante as of 1 January 2023 was 128,312. Therefore, the sample represents approximately 0.91% of the target population in this region. In addition, although a convenience sampling method was used, both private (1) and public (4) schools from the province were included to increase the heterogeneity of the recruited population in terms of school type. Among the participants, 11.2% were in 7th grade, 25.9% were in 8th grade, 28.6% were in 9th grade, 21.5% were in 10th grade, and 12.9% were in 11th grade. Thus, the age range was 12 to 17 years (M = 14.86; DT = 1.41). Table 1 presents the distribution of the sample by sex and age.

The chi-square test for homogeneity showed no statistically significant differences based on gender or school grade (χ^2^ = 9.79; *p* = 0.28). Cramer’s V was 0.06, indicating a small effect size or a weak association.

### 2.2. Instruments 

To assess Executive Function (EF), the Executive Function Questionnaire for Adolescents and Adults was used [55]. This questionnaire is specifically designed for adolescents and adults and consists of 14 items evaluated on a 5-point Likert scale, where 1 corresponds to “definitely not true” and 5 corresponds to “definitely true”. Higher scores indicate greater difficulties in the use of EFs. The questionnaire encompasses two subscales: working memory and inhibitory control. The working memory subscale includes 9 items (e.g., “I have difficulty remembering lengthy instructions”), while the inhibitory control subscale consists of 5 items (e.g., “I sometimes have difficulty stopping an activity I like”). This instrument has demonstrated strong reliability and internal consistency across all of its subscales (working memory α = 0.87; inhibition α = 0.72), as well as for the total score (α = 0.90).

To assess the variable anxiety, the short version of the Depression, Anxiety and Stress Scale (DASS-21) [56] was used. This is a self-reported measure divided into three subscales that evaluate the presence and severity of depression, anxiety, and stress. It employs a Likert-type response scale ranging from 0 to 3 points. Each subscale consists of 7 items, and the total score (ranging from 0 to 21 points) is obtained by summing up the scores of the three subscales. The instrument has demonstrated high reliability and strong internal consistency across all of its subscales (α = 0.86). For this study, only the anxiety subscale was used.

To assess PIU, the Internet Addiction Test (IAT) Spanish version [57] was used. This assessment tool consisting of 20 items rated on a 5-point Likert scale (1 = rarely to 5 = always) evaluates symptoms of Internet addiction through 3 factors: loss of control (inability to control use and neglect of duties), emotional need (satisfaction of emotional needs), and dependence. For Spain, the three factors accounted for 46.68% of the variance. Factor 1 (7 items) explained 18.16% of the variance and measured social and occupational dysfunctions, as well as difficulties with time management. Factor 2 (8 items) explained 15.55% of the variance and measured psychological conflicts related to Internet use. Factor 3 (4 items) explained 13.14% of the variance and measured affective reactions. Item 14 did not load significantly onto any factor. This model demonstrated excellent fit (CFI = 0.989; TLI = 0.987; RMSEA = 0.023; SRMR = 0.040). The total score of the 20 items ranges from 20 to 100, where 20 to 39 suggests controlled Internet use, 40 to 69 represents frequent problems due to Internet use, and scores from 70 to 100 correspond to significant problems related to Internet use. It demonstrates good internal consistency (loss of control α = 0.81, emotional need α = 0.78, dependence α = 0.75, total α = 0.91) [58].

In order to evaluate cyberbullying, the Cyberbullying: Screening of Peer Harassment Questionnaire [59] was used. This is a self-reported measure consisting of two sections that assess different forms of harassment: face-to-face bullying and a broad set of digital harassment behaviors (cyberbullying). The present study focuses specifically on the latter. The questionnaire measures 15 types of harassment behaviors through digital means. Among the behaviors assessed are sending offensive and insulting messages, sexual harassment, spreading photos or videos, password theft, social exclusion on social media, threats, and defamation, among others. It includes three roles, victims, perpetrators, and observers, although only the first two were measured in this study. The instrument employs a triangular response system where the respondent indicates whether they have experienced these harassment behaviors as a victim or perpetrated them as an aggressor over the past year. It consists of 30 items rated on a 4-point Likert scale (0 = never to 3 = always). The test demonstrates adequate internal consistency (α = 0.91), as reported by the authors, and its structure comprises three factors (victim, aggressor, and observer), which explain 40.15% of the variance [60]. The reliability indices for the cyberbullying subscales in the study sample were satisfactory in terms of victimization (α = 0.87) and aggression (α = 0.89).

### 2.3. Procedure

Initial contact was established with the schools to conduct the study, followed by a meeting with the administrative team and the guidance department. During this meeting, the objectives of the study were presented, and both institutional approval and the collaboration of educational staff were requested to facilitate the research process. Subsequently, families (parents, guardians, and legal representatives) were informed about the purpose of the study, and informed consent was obtained for the participation of minors. Of the total recruited sample, only 1.2% (n = 15) of students did not participate in the study because their parents did not provide the signed informed consent form. The questionnaires were administered collectively in the corresponding classrooms, ensuring the anonymity of responses. Throughout this phase, the research team was present at all times to clarify any questions and to ensure that students completed the questionnaires independently and without external influence. This supervision enabled a rigorous and controlled data collection process, ensuring both the reliability of responses and adherence to ethical research standards.

### 2.4. Statistical Analysis

To analyze the relationships between the different variables, the non-parametric Spearman’s correlation coefficient test was used. The interpretation of the results follows these criteria: values between 0.10 and 0.30 indicate a small effect size, values between 0.30 and 0.50 represent a moderate effect size, and values greater than 0.50 indicate a large effect size [61].

To assess the predictive capacity of anxiety, Executive Functions, and cyberbullying with regard to PIU, a binary logistic regression analysis was performed, following a stepwise forward regression procedure based on the Wald statistic. This type of logistic modeling allows for the estimation of the probability of an event occurring (in this case, PIU) in the presence of one or more predictors (the other variables). For this purpose, the odds ratio (OR) statistic was used. All outcome variables were transformed into dichotomous variables based on the cutoff points established by the original authors. For the cyberbullying variables, the cutoff point was 16; thus, a score equal to or greater than 16 was coded as “cybervictimization/cyberbullying”, and any score below 16 was coded as “no cybervictimization/no cyberbullying”. The same procedure was applied to the PIU variable, with a cutoff point of 40.

All analyses were conducted using the statistical software SPSS version 26.0.

## 3. Results

### 3.1. Correlations

First, a correlation analysis was conducted among the variables using Spearman’s correlation coefficient.

The results show that the relationship between the variables is significant and positive in all cases.

Regarding PIU, a moderate relationship was observed with Executive Functions (0.30–0.33) and anxiety (0.31, 0.33), while the correlation with cyberbullying variables was small (0.19–0.26). Concerning cybervictimization, a moderate relationship was found with Executive Functions (0.31–0.37) and anxiety (0.36), whereas its correlation with PIU was small (0.24–0.30). Cyberbullying showed a small relationship with inhibitory control (0.22) and PIU components (0.19–0.23), while its correlation with working memory and anxiety was low (Table 2).

### 3.2. Logistic Regression

#### 3.2.1. Problematic Internet Use Based on Executive Functions, Anxiety, Cyberbullying, and Cybervictimization

From the data analysis, it was possible to create five predictive models for the explanation of PIU. The logistic regression analyses indicate that PIU is explained by Executive Functions (EFs), anxiety, cyberbullying, and cybervictimization among students. The two models in which Executive Functions (working memory and inhibition) are the predictor variables correctly classify 67.6% of cases (χ^2^ = 27.07; *p* < 0.001) and 68.4% of cases (χ^2^ = 38.89; *p* < 0.001), with Nagelkerke’s R^2^ values of 0.078 and 0.111, respectively. The anxiety-based model correctly classifies 63.8% of cases (χ^2^ = 42.16; *p* < 0.001), with a goodness-of-fit value (Nagelkerke’s R^2^) of 0.07. Finally, the PIU models based on cybervictimization and cyberbullying correctly classify 60.1% of cases (χ^2^ = 56.72; *p* < 0.001) and 60.1% of cases (χ^2^ = 31.62; *p* < 0.001), with Nagelkerke’s R^2^ values of 0.07 and 0.04, respectively.

The odds ratios (ORs) of the models indicate that students are 1.07 and 1.15 times more likely to exhibit PIU for each one-unit increase in their working memory and inhibition, respectively. Additionally, adolescents have a 1.11 times higher probability of PIU for each one-unit increase in anxiety levels. Likewise, students are 1.14 and 1.20 times more likely to present PIU as their cybervictimization and cyberbullying scores increase by one unit, respectively (see Table 3).

#### 3.2.2. Cybervictimization Based on EFs, Anxiety, and PIU

From the data analysis, it was possible to generate six predictive models that explain cybervictimization. The logistic regression analysis shows that cybervictimization is explained by Executive Functions (EFs), anxiety, and PIU in students. The two models with EFs (working memory and inhibition) as predictor variables correctly classified 63.2% of the cases (χ^2^ = 31.89; *p* < 0.001) and 63.4% of the cases (χ^2^ = 45; *p* < 0.001), with goodness-of-fit values (Nagelkerke R^2^) of 0.08 and 0.12, respectively. The explanatory model using anxiety correctly classified 64.8% of the cases (χ^2^ = 56.78; *p* < 0.001), with a goodness-of-fit value (Nagelkerke R^2^) of 0.10. Finally, the models of cybervictimization using PIU (loss of control, emotional need, and dependence) correctly classified 61.7% (χ^2^ = 40.65; *p* < 0.001), 63.5% (χ^2^ = 63.32; *p* < 0.001), and 63% (χ^2^ = 46.34; *p* < 0.001) of the cases, with goodness-of-fit values of 0.05, 0.08, and 0.06 (Nagelkerke R^2^), respectively.

The odds ratios (ORs) of the models indicate that students have 1.07 and 1.16 times higher probability of being cybervictims for each one-unit increase in their working memory and inhibition, respectively. Additionally, adolescents have a 1.14 times higher probability of experiencing cybervictimization for each one-unit increase in their anxiety. PIU also explained cybervictimization. Specifically, students have 1.08, 1.11, and 1.17 times higher probability of experiencing cybervictimization for each one-unit increase in their PIU scores (loss of control, emotional need, and dependence, respectively) (see Table 4).

#### 3.2.3. Cyberbullying Based on EFs, Anxiety, and PIU

From the data analysis, it was possible to create six predictive models for the explanation of cyberbullying. The logistic regression analyses indicate that cyberbullying is explained by Executive Functions (EFs) and PIU among students. No statistically significant results were found for anxiety or working memory. The model with inhibition as the predictor variable correctly classified 62.7% of the cases (χ^2^ = 31.89; *p* < 0.001), with a goodness-of-fit value (Nagelkerke R^2^) of 0.04. The models of cyberbullying using PIU (emotional need, loss of control, and dependence) correctly classified 64.9% (χ^2^ = 37.05; *p* < 0.001), 64.2% (χ^2^ = 31.75; *p* < 0.001), and 64.2% (χ^2^ = 28.27; *p* < 0.001) of the cases, with goodness-of-fit values of 0.05, 0.04, and 0.04 (Nagelkerke R^2^), respectively.

The odds ratios (ORs) of the models indicate that students are 1.09 times more likely to engage in cyberbullying for each one-unit increase in their inhibition. Additionally, students have 1.07, 1.08, and 1.13 times higher probability of experiencing cybervictimization for each one-unit increase in their PIU scores (loss of control, emotional need, and dependence, respectively) (see Table 5).

## 4. Discussion

The main objective of this research was to analyze the risks associated with Internet use during adolescence and their relationship with Executive Functions (EFs) and anxiety. More specifically, the interaction between three key phenomena was examined—Problematic Internet Use (PIU), cybervictimization, and cyberbullying—and their link to these psychological variables, with the aim of better understanding the factors that may influence the development of risky behaviors in digital environments.

As a preliminary step, the distribution of the sample by sex and age was analyzed, and no statistically significant differences were found. This aligns with findings reported by other researchers [62,63,64].

The first hypothesis (H1) proposed that Problematic Internet Use (PIU) would be more frequent in adolescents with lower development of Executive Functions, higher levels of anxiety, and a history of cyberbullying and cybervictimization. The results confirmed this hypothesis, both through significant positive correlations and regression analyses, in which five predictive models were developed to explain PIU based on Executive Functions (EFs), anxiety, cyberbullying, and cybervictimization.

The findings align with previous research that has identified an association between deficits in inhibitory control and PIU in adolescents, as demonstrated through multiple linear regression analyses [24]. Similarly, systematic reviews have indicated that certain psychological and physiological variables, including anxiety, may be related to the development of PIU [49]. Furthermore, recent studies have shown a significant relationship between PIU, cyberbullying, and cybervictimization, suggesting that interaction dynamics in digital environments may act as a risk factor for the development of this behavior [50,53].

Difficulties in Executive Functions (EFs) can lead to a pattern of PIU, where a lack of self-control, planning, and emotional regulation fosters greater dependence on online interactions [49]. This tendency is reinforced by impulsivity and difficulties in managing time and priorities, which can lead individuals to neglect their academic, work, or social responsibilities [50].

In this context, anxiety plays a key role, as many individuals turn to the Internet as a maladaptive emotional regulation strategy, seeking temporary relief from their distress through excessive consumption of digital content or interaction on social media [52]. However, this type of coping mechanism can perpetuate Internet dependency, creating a vicious cycle in which avoiding anxiety through excessive use of electronic devices ultimately exacerbates the problem.

Sense of belonging plays a central role during adolescence and is essential for understanding the difficulties related to psychological development and school adjustment. A study [65] examined the relationship between belonging at school and belonging on social media, finding that both variables were negatively correlated with psychological maladjustment and academic performance, with social media addiction acting as a mediating factor. This impact on adolescents’ psychological health and school adaptation is crucial for understanding how the phenomenon of cyberbullying emerges. Moreover, disruptions in sense of belonging may also help explain why some adolescents seek refuge in online environments or become involved in harmful digital behaviors, reinforcing the patterns observed in Problematic Internet Use.

Furthermore, the impact of PIU can be observed in both victims and aggressors. Victims, feeling rejected or excluded in face-to-face social environments, may use the Internet as an escape mechanism, seeking refuge in virtual communities or online relationships that provide them with a sense of belonging. On the other hand, aggressors, in addition to using digital platforms to harass their victims, often exhibit higher levels of impulsivity and low inhibitory control, which facilitates the repetition of aggressive behaviors without adequate reflection on the consequences of their actions [20].

These elements collectively reinforce the need for interventions aimed at strengthening Executive Functions, promoting strategies that enable more conscious and adaptive use of the Internet, as well as effective tools for managing anxiety and preventing cyberbullying. Understanding these mechanisms is essential for designing prevention and intervention programs that reduce the negative effects of PIU and promote a healthy balance in the use of digital technologies.

The second hypothesis (H2) proposed that students would have a higher probability of experiencing cybervictimization when they exhibited difficulties in Executive Functions (EFs), high levels of anxiety, and PIU. This relationship is explained by various psychological and behavioral factors that may increase the vulnerability of young people in digital environments.

First, young people with higher anxiety may be more likely to become victims of cyberbullying, as they tend to show signs of emotional insecurity that can be perceived and exploited by aggressors. Additionally, lower competence in social skills may hinder their ability to defend themselves or seek help, increasing their helplessness in the face of online attacks. Furthermore, anxiety can lead to increased time spent in the digital world, thereby raising the likelihood of encountering risky situations [20].

On the other hand, difficulties in Executive Functions (EFs) can also contribute to cybervictimization. Impulsivity and low inhibitory control may lead to more intense reactions to online provocations, which could prolong and escalate conflicts in digital environments. Additionally, poor decision making may result in interactions with strangers or exposure on digital platforms without adequately assessing the potential consequences [50].

These factors reinforce the need for interventions focused on emotional management, the development of social skills, and the strengthening of Executive Functions aiming to reduce young people’s vulnerability to cybervictimization and promote safer and more conscious use of the Internet.

The third hypothesis (H3) proposed that the probability of engaging in cyberbullying would be related to difficulties in Executive Functions (EFs), high levels of anxiety, and PIU. The results supported the proposed hypothesis, aligning with previous findings [54]. Likewise, prior research has demonstrated the relationship between Executive Functions (EFs), inhibition capacity, self-control, and problem-solving skills, which are key factors in participation in cyberbullying dynamics.

Despite these findings, this study has certain limitations that should be considered in future research. Due to its cross-sectional design, it is not possible to establish causal relationships between the analyzed variables. To address this limitation, it would be advisable to conduct prospective longitudinal studies. Likewise, it would be beneficial to include a random sampling method and to identify the reasons why some parents or legal guardians did not provide consent for their children to participate in the study in order to address these issues in future implementation. It would also be appropriate to apply the study to primary education to gain deeper insight into how the use of new technologies and their associated risks emerge at an early age. In addition, it would be valuable to explore the perspectives and knowledge that teachers and parents have regarding the phenomena studied. Finally, to ensure that the results more accurately reflect the national reality, it would be necessary to carry out the evaluation in a greater number of provinces, including schools located in both urban and rural areas.

Beyond the aspects mentioned, we think it would be of interest for future lines of research to focus on the traumatic aspect of cyberbullying, given that the effect of trauma on the brain has been extensively studied. Various authors have shown that individuals who have undergone traumatic experiences often present alterations in the dorsal prefrontal networks, suggesting deficits in executive control, particularly in response inhibition, working memory, attention regulation, cognitive flexibility, and stimulus response inhibition, which tend to worsen in emotionally charged or trauma-related contexts [66,67,68]. This line of inquiry could help to better understand the impact of trauma on executive functioning, both in victims and in aggressors.

However, the results obtained have practical implications in the educational, mental health, and even family domains, emphasizing the importance of educating and raising awareness among the various agents who accompany young people at this stage of life about how the virtual world operates.

With regard to adolescents, in the educational context, it is recommended to include strategies for self-regulation and critical thinking, promote group work, and strengthen the early detection of psychological difficulties [45]. In the clinical setting, these findings can refine therapeutic interventions and improve emotional regulation in adolescents.

Families can benefit from awareness campaigns fostering open dialogue about Internet use and promoting healthy digital habits [69,70,71,72]. Although it may be difficult for some adults to understand, for young people, technology constitutes a bridge between the real and the virtual world. Building upon emotional context, another critical factor to consider is the fear of missing out (FoMO), which directly affects adolescents’ well-being today. A previous study [73] investigated the relationship between peer exclusion from WhatsApp classmate groups, FoMO, and emotional symptoms. The findings showed that exclusion from online classmate groups was positively associated with both FoMO and emotional distress. Importantly, FoMO served as a mediating variable, meaning that adolescents who experienced greater online exclusion reported higher levels of FoMO, which in turn was linked to more emotional symptoms. The study highlights how cyber exclusion, also a form of cyberbullying, can negatively affect emotional well-being during early adolescence by increasing the apprehension of being disconnected from peers. Something as simple as knowing whether a child or student is an active member of WhatsApp groups can provide information about a possible situation of marginalization, serving as a modern equivalent of the sociogram and helping to address cases of cyberbullying, which, due to their invisible nature, often go unnoticed.

It is necessary to raise awareness among parents regarding the permissiveness with which smartphones are currently provided at early ages and the little or no supervision that follows. The importance lies in the fact that these devices provide access to unlimited interaction, something previous generations never experienced. In the past, in our social relationships, we received positive or negative feedback from a small group of peers. Now, however, with any content we share through social networks, we are exposed to the opinions of hundreds of peers. Moreover, the way social media platforms operate allows peers to make statements without being aware of the harm they may cause to the recipient, as they do not see their reaction [74]. This means that greater harm can be inflicted without remorse [75].

In the case of teachers, while they do not have access to supervise students’ use of social media, they can be trained to teach their students to identify the different roles adopted by aggressors, victims, and bystanders in situations of abuse and thus help prevent such behaviors [76].

Overall, campaigns and intervention projects aimed at the prevention and management of PIU and its associated risks could bring significant benefits to society.

## Figures and Tables

**Table 1:** Sample distribution by sex and school grade.

			School Grade					Total
			7th	8th	9th	10th	11th	
Sex	Male	N	58	141	164	130	67	560
		%	5.0	12.1	14.1	11.2	5.8	48.1
	Female	N	72	160	169	120	83	604
		%	6.2	13.7	14.5	10.3	7.1	51.9
Total		N	130	301	333	250	150	1164
		%	11.2	25.9	28.6	21.5	12.9	100.0

Note: 7th to 10th grades correspond to *Educación Secundaria Obligatoria* (ESO) and 11th grade corresponds to *1st of Bachillerato* in the Spanish educational system.

**Table 2:** Correlation between different variables.

	1	2	3	4	5	6	7	8	9
1. EFwm	1								
2. EFic	0.73 **	1							
4. Anx	0.44 **	0.36 **	0.44 **	1					
5. Cv	0.31 **	0.37 **	0.36 **	0.36 **	1				
6. Cb	0.09 *	0.22 **	0.15 **	0.09 *	0.46 **	1			
7. PIUlc	0.26 **	0.30 **	0.30 **	0.31 **	0.24 **	0.20 **	1		
8. PIUen	0.33 **	0.33 **	0.35 **	0.33 **	0.30 **	0.23 **	0.67 **	1	
9. PIUdep	0.30 **	0.30 **	0.32 **	0.26 **	0.26 **	0.19 **	0.69 **	0.70 **	1

Note. EFwm = Executive Function (working memory), EFic = Executive Function (inhibitory control), Anx = anxiety, Cv = cybervictimization, Cb = cyberbullying, PIUlc = Problematic Internet Use (loss of control), PIUen = Problematic Internet Use (emotional need), PIUdep = Problematic Internet Use (dependence), * correlation is significant at the 0.05 level (two-tailed), ** correlation is significant at the 0.01 level (two-tailed).

**Table 3:** Binary logistic regression of the probability of presenting PIU based on EFs, anxiety, cyberbullying, and cybervictimization.

	B	S.E.	Wald	*p*	OR	C.I. 95%
EF working memory	0.07	0.01	25.51	<0.001	1.07	1.04–1.10
Constant	−2.35	0.32	51.61	<0.001	0.09	
EF inhibition	0.14	0.02	35.03	<0.001	1.15	1.10–1.21
Constant	−2.83	0.36	60.13	<0.001	0.05	
Anxiety	0.10	0.02	39.97	<0.001	1.11	1.07–1.14
Constant	−1.72	0.21	63.92	<0.001	0.17	
Cybervictimization	0.13	0.02	40.97	<0.001	1.14	1.09–1.18
Constant	−0.45	0.07	35.01	<0.001	0.63	
Cyberbullying	0.18	0.04	19.16	<0.001	1.20	1.11–1.30
Constant	−0.29	0.07	18.39	<0.001	0.75	

Note: B = coefficient; S.E. = standard error; *p* = probability; OR = odds ratio; C.I. 95% = confidence interval at 95%.

**Table 4:** Binary logistic regression of the probability of experiencing cybervictimization based on EFs, anxiety, and PIU.

	B	S.E.	Wald	*p*	*OR*	C.I. 95%
EF working memory	0.07	0.01	28.45	<0.001	1.07	1.05–1.10
Constant	−1.14	0.30	14.26	<0.001	0.32	
EF inhibition	0.15	0.02	39.16	<0.001	1.16	1.11–1.22
Constant	−1.57	0.33	23.20	<0.001	0.21	
Anxiety	0.13	0.02	47.65	<0.001	1.14	1.09–1.18
Constant	−1.05	0.22	21.84	<0.001	0.35	
PIU loss of control	0.08	0.01	38.02	<0.001	1.08	1.06–1.11
Constant	−0.83	0.21	15.5	<0.001	0.44	
PIU emotional need	0.11	0.01	54.61	<0.001	1.11	1.08–1.14
Constant	−1.03	0.20	26.02	<0.001	0.36	
PIU dependence	0.16	0.03	41.39	<0.001	1.17	1.12–1.23
Constant	−0.71	0.18	15.02	<0.001	0.49	

Note: B = coefficient; S.E. = standard error; *p* = probability; OR = odds ratio; C.I. 95% = confidence interval at 95%.

**Table 5:** Binary logistic regression of the probability of perpetrating cyberbullying based on EFs, anxiety, and PIU.

	B	S.E.	Wald	*p*	*OR*	C.I. 95%
EF inhibition	0.08	0.02	14.25	<0.01	1.09	1.04–1.13
Constant	−1.65	0.32	26.98	<0.01	0.19	
PIU loss of control	0.07	0.01	30.88	<0.01	1.07	1.05–1.10
Constant	−1.70	0.21	62,79	<0.01	0.18	
PIU emotional need	0.07	0.01	35.63	<0.01	1.08	1.05–1.10
Constant	−1.64	0.19	73.53	<0.01	0.19	
PIU dependence	0.12	0.02	27.65	<0.01	1.13	1.08–1.18
Constant	−1.48	0.18	65.34	<0.01	1.12	

Note: B = coefficient; S.E. = standard error; *p* = probability; OR = odds ratio; C.I. 95% = confidence interval at 95%.

## Data Availability

Due to the explicit prohibition by the ethics committee of the disclosure of minors’ data, it is not possible to provide such data.
